# Genomic Analysis of *Pseudomonas* sp. Strain SCT, an Iodate-Reducing Bacterium Isolated from Marine Sediment, Reveals a Possible Use for Bioremediation

**DOI:** 10.1534/g3.118.200978

**Published:** 2019-03-25

**Authors:** Masafumi Harada, Kohei Ito, Nobuyoshi Nakajima, Shigeki Yamamura, Masaru Tomita, Haruo Suzuki, Seigo Amachi

**Affiliations:** * Institute for Advanced Biosciences, Keio University, Tsuruoka, Yamagata, Japan; † Faculty of Environment and Information Studies, Keio University, Fujisawa, Kanagawa, Japan; ‡ Center for Environmental Biology and Ecosystem Studies, and; § Center for Regional Environmental Research, National Institute for Environmental Studies, Tsukuba, Ibaraki, Japan, and; ** Graduate School of Horticulture, Chiba University, Matsudo City, Chiba, Japan

**Keywords:** marine sediment, iodate-reducing bacterium, strain SCT, *Pseudomonas stutzeri*, genome analysis, comparative genomics, phylogeny, gene conservation

## Abstract

Strain SCT is an iodate-reducing bacterium isolated from marine sediment in Kanagawa Prefecture, Japan. In this study, we determined the draft genome sequence of strain SCT and compared it to complete genome sequences of other closely related bacteria, including *Pseudomonas stutzeri*. A phylogeny inferred from concatenation of core genes revealed that strain SCT was closely related to marine isolates of *P. stutzeri*. Genes present in the SCT genome but absent from the other analyzed *P. stutzeri* genomes comprised clusters corresponding to putative prophage regions and possible operons. They included *pil* genes, which encode type IV pili for natural transformation; the *mer* operon, which encodes resistance systems for mercury; and the *pst* operon, which encodes a Pi-specific transport system for phosphate uptake. We found that strain SCT had more prophage-like genes than the other *P. stutzeri* strains and that the majority (70%) of them were SCT strain-specific. These genes, encoded on distinct prophage regions, may have been acquired after branching from a common ancestor following independent phage transfer events. Thus, the genome sequence of *Pseudomonas* sp. strain SCT can provide detailed insights into its metabolic potential and the evolution of genetic elements associated with its unique phenotype.

The genus *Pseudomonas* consists of gram-negative bacteria that inhabit a wide variety of environments, including the sea, soil, rhizospheres of plants, and the human microbiome (Aagot *et al.* 2001; Tekorienė 2008). Depending on the habitat, they employ various metabolic strategies, such as denitrification, nitrogen fixation, and metal reduction (Lovley 1993; Lloyd 2003). *Pseudomonas* spp. have thus been applied in bioremediation, for the removal or detoxification of environmental pollutants (Wasi *et al.* 2013). A 16S rRNA phylogenetic analysis split *Pseudomonas* into several subgroups (Anzai *et al.* 2000); one of them includes *Pseudomonas stutzeri*, *Pseudomonas balearica*, and *Pseudomonas luteola*. *P. stutzeri* is widespread and occupies diverse ecological niches, as listed exhaustively by Lalucat *et al.* (2006).

Iodine is an essential element for humans, as it is the main component of thyroid hormone and its deficiency causes goiter (Zava and Zava 2011). Historically, industrial iodine was extracted from ashes after burning seaweed, suggesting its abundance in marine environments (Zava and Zava 2011). Nowadays, iodine is taken up by ingesting marine products. At the same time, iodine in the oceans is involved in microbial metabolism (Winchester and Duce 1967; Amachi 2008). Strain SCT is a bacterium isolated from the slurry/sediment in Sagami Bay, Kanagawa Prefecture, Japan, by enrichment culture of iodate-reducing bacteria (Amachi *et al.* 2007). A phylogenetic analysis based on 16S rRNA gene sequencing indicated that strain SCT was most closely related to *Pseudomonas stutzeri* (Amachi *et al.* 2007).

Results by Amachi *et al.* (2007) suggest “SCT is a dissimilatory iodate-reducing bacterium and that its iodate reductase is induced by iodate under anaerobic growth conditions”. However, whereas the iodate-reducing activity of strain SCT has been extensively studied, its other potential functions remain largely unknown. The purpose of this study was to gain insights into the evolution and functional potential of strain SCT. To this end, we performed whole-genome shotgun sequencing of strain SCT and comparative genomic analyses with closely related bacteria including *P. stutzeri*.

## Materials and Methods

### Software

Bioinformatics analyses were conducted using Python version 3.5.5 and its molecular biology package Biopython version 1.72 (Cock *et al.* 2009). Statistical computing was implemented using R version 3.4.3, available at https://www.r-project.org/ (R Development Core Team 2008).

### Sequence data

To compare strain SCT to the genome of *Pseudomonas* sp., we used 10 *P. stutzeri* and one *P. balearica* strains for which complete genome sequences were available through public databases. They included *P. stutzeri* Cr(VI)- and nitrate-reducing strain RCH2 (Chakraborty *et al.* 2017), naphthalene-degrading strain 19SMN4 (Rosselló-Mora *et al.* 1994), naphthalene-degrading strain CCUG 29243 (equivalent to AN10) (Brunet-Galmés *et al.* 2012), petroleum-emulsifying strain SLG510A3-8 (Hu *et al.* 2015), nitrogen-fixing and rhizosphere-associated strain DSM 4166 (Yu *et al.* 2011), type strain CGMCC 1.1803 (ATCC 17588) (Chen *et al.* 2011), nitrogen-fixing root-associated strain A1501 (Yan *et al.* 2008), highly transformable strain 28a24 (Smith *et al.* 2014), exopolysaccharide-producing strain 273 (Wu *et al.* 2017), natural transformation model strain DSM 10701 (JM300) (Busquets *et al.* 2012), and *P. balearica* DSM 6083T (Bennasar-Figueras *et al.* 2016). Based on a previous phylogenetic study (Özen and Ussery 2012), *Pseudomonas mendocina* ymp was used as an outgroup to root the phylogenetic tree. Genome sequence data in GenBank format (Benson *et al.* 2017) were retrieved/downloaded on 2018-02-24 from the National Center for Biotechnology Information (NCBI) reference sequence (RefSeq) database (Pruitt *et al.* 2007) using a set of scripts obtained from https://github.com/kblin/ncbi-genome-download. The final data set consisted of 13 *Pseudomonas* strains as shown in Table 1.

**Table 1:** Genomic features of *Pseudomonas* strains analyzed

Accession*^a^*	Organism	Isolation source	Size (Mb)	*^b^*GC (%)	*^c^*CDS	*^d^*Phage	*^e^*Conserved
This study	*Pseudomonas* sp. SCT	Marine sediment	4.79	62.5	4520	184	4520
GCF_000327065.1	*Pseudomonas stutzeri* RCH2	Cr(VI)-contaminated aquifer	4.58	62.5	4353	43	3919
GCF_000661915.1	*Pseudomonas stutzeri* 19SMN4	Marine sediment	4.73	62.3	4533	109	3678
GCF_000267545.1	*Pseudomonas stutzeri* CCUG 29243 (=AN10)	Marine sediment	4.71	62.7	4417	95	3578
GCF_000195105.1	*Pseudomonas stutzeri* SLG510A3-8	Oil-contaminated soil	4.65	64.0	4342	33	3547
GCF_001038645.1	*Pseudomonas stutzeri* DSM 4166	*Sorghum nutans* rhizosphere	4.69	64.0	4427	0	3560
GCF_000219605.1	*Pseudomonas stutzeri* CGMCC 1.1803	Clinical specimen	4.55	63.9	4242	23	3473
GCF_000013785.1	*Pseudomonas stutzeri* A1501	Rice paddy soil	4.57	63.9	4270	31	3461
GCF_000590475.1	*Pseudomonas stutzeri* 28a24	Soil	4.73	60.6	4317	27	2367
GCF_001648195.1	*Pseudomonas stutzeri* 273	Marine sediment	5.03	60.3	4687	77	2580
GCF_000279165.1	*Pseudomonas stutzeri* DSM 10701	Soil	4.17	63.2	3931	0	2463
GCF_000818015.1	*Pseudomonas balearica* DSM 6083	Wastewater treatment plant	4.38	64.0	4126	52	2522
GCF_000016565.1	*Pseudomonas mendocina* ymp	PCP-contaminated soil	5.07	64.7	4714	68	1612

a NCBI RefSeq accession number.

b Percentage of G and C in the nucleotide sequence, defined as 100 × (G+C)/(A+T+G+C).

c Number of protein-coding DNA sequences (CDSs).

d Number of CDSs in phage-like regions predicted using PHASTER.

e Number of SCT genes conserved in each genome inferred using LS-BSR.

### Genome sequencing, assembly, and annotation of strain SCT

Strain SCT was grown aerobically in Lysogeny broth (LB) medium. Genomic DNA was extracted using a DNeasy blood and tissue kit (Qiagen, Hilden, Germany). According to the manufacturer’s protocols, an Illumina paired-end library (with an average insert size of 550 bp) was prepared, and whole-genome sequencing was performed using an Illumina MiSeq sequencing platform (Illumina, San Diego, CA) at the National Institute for Environmental Studies. The sequencer produced 1,084,678 paired-end reads (2 × 300 bp).

The paired-end reads were processed as follows: PhiX contaminations were removed using bbduk (https://sourceforge.net/projects/bbmap/). bbduk was run with the following parameters: “ref= phiX.fasta k=31 hdist=1” to remove all reads that had a 31-mer match to phiX (NCBI RefSeq accession: NC_001422), allowing one mismatch. After that, adapters were removed and reads were trimmed using Trimmomatic version 0.36 (Bolger *et al.* 2014) by typing “java -jar trimmomatic-0.36.jar PE -phred33 input_forward.fq input_reverse.fq output_forward_paired.fq output_forward_unpaired.fq output_reverse_paired.fq output_reverse_unpaired.fq ILLUMINACLIP:$adaptors:2:30:10 SLIDINGWINDOW:4:30 LEADING:3 TRAILING:3 MINLEN:100” (http://www.usadellab.org/cms/?page=trimmomatic). *De novo* assembly was conducted using SPAdes version 3.9.0 (Bankevich *et al.* 2012) with the following parameters “–careful–cov-cutoff auto”. After low-coverage contigs were disregarded, the resulting assembly consisted of 35 contigs containing 4,791,932 bp, with a G+C content of 62.5% and sequencing coverage of 56.5x.

Protein-coding DNA sequences (CDSs) were predicted and functional annotations (gene and product names) were assigned using Prokka version 1.11, which coordinates a suite of existing bioinformatics tools and databases for annotation of prokaryotic genome sequences (Seemann 2014), incorporating Prodigal (Hyatt *et al.* 2010), BLAST+ (Altschul *et al.* 1997), and HMMER (http://hmmer.org/). Prokka was run with the following parameters: “–kingdom Bacteria–compliant” (https://github.com/tseemann/prokka). We then performed similarity searches of all the predicted protein sequences against the UniRef90 sequence database (UniRef90 Release 2016_08 consisting of 44,448,796 entries) using the BLASTP program with an E-value cutoff of 1e-5, and assigned functional annotations from the most similar (best hit) protein sequences. BlastKOALA (Kanehisa *et al.* 2016) was used to assign KEGG Orthology identifiers to the protein sequences obtained by BLAST searches, for which taxonomy group information on “Bacteria” and the KEGG Genes database of “family_eukaryotes + genus_prokaryotes” were selected at https://www.kegg.jp/blastkoala/.

### Search for mobile genetic elements

Mobile genetic elements such as phages were searched in the *Pseudomonas* genomes. The PHASTER search tool (Arndt *et al.* 2017) was used to identify putative prophage regions in the 13 *Pseudomonas* genomes analyzed. A new search of phage sequences in the SCT genome was performed at http://phaster.ca/, and pre-calculated results for the other 12 *Pseudomonas* genomes were obtained from http://phaster.ca/submissions.

### Phylogenetic analysis

To infer phylogenetic relationships among the 13 *Pseudomonas* strains, we used single-copy core genes, which are shared by all genomes and contain only a single copy from each genome (and thus contain orthologs, but not paralogs). The core genes were built using the Roary pan-genomic analysis pipeline (Page *et al.* 2015) with a default parameter. All nucleotide alignments of the core genes were done in MAFFT (Katoh *et al.* 2002) and then concatenated by Roary. We used RAxML version 8.2.11 (Stamatakis 2014) for maximum likelihood-based inference of a phylogenetic tree on the concatenated sequence alignment under the GTR+CAT model. RAxML was run as follows: “raxmlHPC-PTHREADS -f a -x 12345 -p 12345 -# 100 -m GTRCAT -s ./core_gene_alignment.phy -n outfile”. The resulting tree was drawn using FigTree version 1.4.3, available at http://tree.bio.ed.ac.uk/software/figtree/.

### Gene conservation analysis

To assess the conservation of SCT protein-coding genes in the other *Pseudomonas* genomes, we used the large-scale blast score ratio (LS-BSR) (Sahl *et al.* 2014). Briefly, the LS-BSR pipeline performed a TBLASTN search using the protein sequence of strain SCT as a query and the whole nucleotide sequence of each of the *Pseudomonas* strains as a database, and calculated the BSR. The obtained BSR value ranged from 0 (no sequence similarity) to 1 (maximal sequence similarity) and was used as a measure of the degree of conservation of SCT genes in the other *Pseudomonas* genomes. The ’$prefix_bsr_matrix.txt’ file contains the BSR value for each gene in each genome, and the ’$prefix_dup_matrix.txt’ file was used to determine gene presence and absence in each genome (https://github.com/jasonsahl/LS-BSR).

### Data availability

The draft genome sequence of *Pseudomonas* sp. strain SCT has been deposited at GenBank/EMBL/DDBJ under BioProject number PRJDB5044, BioSample number SAMD00059319, and accession number BDJA00000000 (accession range: BDJA01000001-BDJA01000035). The version described in this paper is the first version, BDJA01000000. The raw reads have been deposited in the DDBJ Sequence Read Archive (DRA) under Submission DRA007938, Experiment DRX156286, and Run DRR165667.

Supplementary Table S1 has been deposited via the GSA Figshare portal. The Python scripts used in this study are available through the Github repository https://github.com/haradama/PSCT. Supplemental material available at Figshare: https://doi.org/10.25387/g3.7829321.

## Results and Discussion

### Phylogeny

**Figure 1:**
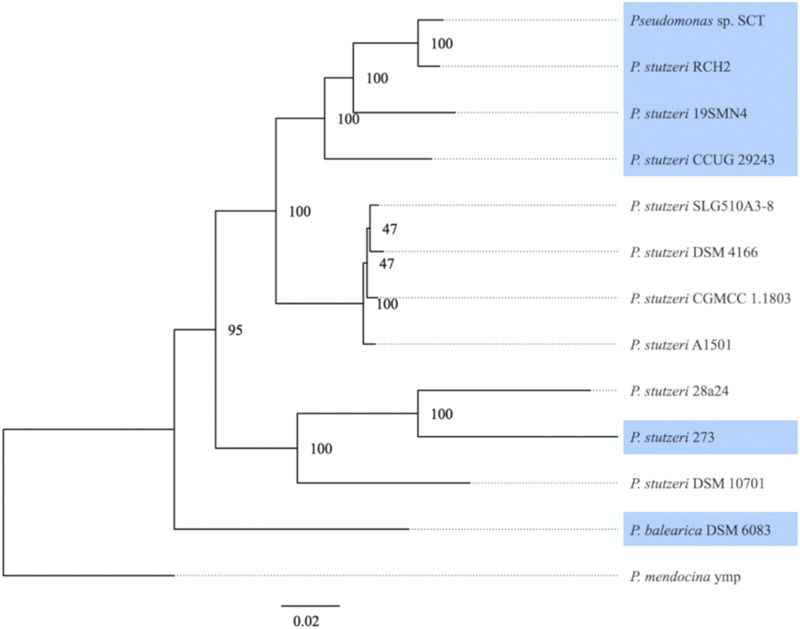
Maximum likelihood tree obtained from a concatenated nucleotide sequence alignment of core genes for the 13 *Pseudomonas* strains. The horizontal bar at the base of the figure represents 0.02 substitutions per nucleotide site. Bootstrap support values for each of the branches of the tree are indicated based on 100 bootstrap replicates. Blue represents strains isolated from water environments such as marine sediments.

An accurate phylogenetic tree of a group of organisms provides a valid inference of its evolutionary history, gene gain, and loss events (Song *et al.* 2017). The subgroups of the genus *Pseudomonas*, including the *P. stutzeri* group, have been defined based on phylogenetic analyses of 16S rRNA gene sequences (Anzai *et al.* 2000). Recent studies have demonstrated that 16S rRNA gene sequences do not contain enough phylogenetic signals to distinguish closely related bacteria such as strains within the same species (Fox *et al.* 1992; Özen and Ussery 2012). To attain higher phylogenetic resolution, we inferred the phylogenetic relationship of SCT and other *Pseudomonas* strains based on 53 conserved core genes. Figure 1 shows the maximum likelihood phylogenetic tree based on the concatenated nucleotide sequence alignment of core genes. Core genome phylogeny with 100% bootstrap support indicated that strain SCT and *P. stutzeri* strain RCH2 (Chakraborty *et al.* 2017) formed a monophyletic group or clade that included also, in decreasing order of relevance, *P. stutzeri* strain 19SMN4 (Rosselló-Mora *et al.* 1994) and *P. stutzeri* strain CCUG 29243 (Brunet-Galmés *et al.* 2012). Results suggest that SCT belongs to *P. stutzeri* and is the sister strain to *P. stutzeri* strain RCH2.

The earliest branching lineage in this tree (Figure 1) was *P. balearica*, followed by the *P. stutzeri* clade. The latter contained three subgroups: the first one comprised strains SCT, RCH2, 19SMN4, and CCUG 29243; the second comprised strains SLG510A3-8, DSM 4166, CGMCC 1.1803, and A1501; and the third comprised strains 28a24, 273, and DSM 10701. A previous study revealed that distinct subgroups for the *P. stutzeri* clade could be accredited to ecotype status resulting from niche-specific adaptations; accordingly, the first subgroup contained marine isolates and the second subgroup contained soil/sludge isolates (Sharma *et al.* 2015). Given the primary niche of the first subgroup strains (SCT, RCH2, 19SMN4, and CCUG 29243), and a comparison to other strains in the *P. stutzeri* clade (Table 1), this phylogeny suggests that adaptation to marine/aquifer and soil/rhizosphere environments might have evolved after divergence of these subgroups from a common ancestor.

### Genome features

Genome features can reflect not only phylogenetic positions but also lifestyles or ecological niches, as indicated by free-living soil bacteria with large G+C-rich genomes and obligatory intracellular symbionts with small G+C-poor genomes (Dutta and Paul 2012). Table 1 shows the genome features (size, G+C content, and CDS number) of the 13 *Pseudomonas* strains included in this analysis. Genome size ranged from 4.17 Mb to 5.07 Mb with a median of 4.69 Mb, G+C content ranged from 60.3 to 64.7% with a median of 63.2%, and CDS numbers ranged from 3,931 to 4,714 with a median of 4,353. The genome features of strain SCT were thus in line with those of other *Pseudomonas* strains. The G+C content for the first subgroup (ranging from 62.3 to 62.7%) in our phylogenetic tree (Figure 1), was lower than that of the second subgroup (ranging from 63.9 to 64.0%).

In contrast to previous studies showing a positive correlation between genome size and G+C content for sequenced bacterial genomes (McCutcheon *et al.* 2009; Dutta and Paul 2012), a correlation between genome size and G+C content for the 13 *Pseudomonas* strains used in this study was weakly negative (Pearson’s product-moment correlation coefficient, r = -0.34 and *p*-value = 0.25; Spearman’s rank correlation coefficient, rho = -0.33 and *p*-value = 0.27).

### Mobile genetic elements

Horizontal transfer of DNA occurs generally via three different mechanisms: conjugation, transformation, and transduction (Kwong *et al.* 2000). It is now widely accepted that mobile genetic elements, such as plasmids and phages, contribute to the evolution of bacteria and the spreading of virulence and drug resistance in microbial communities (Frost *et al.* 2005).

Putative prophage regions have been detected in the genomes of *P. stutzeri* strains CCUG 29243 (Brunet-Galmés *et al.* 2012) and DSM 10701 (Busquets *et al.* 2012), as well as *P. balearica* DSM 6083 (Bennasar-Figueras *et al.* 2016). The PHASTER phage search tool identified six putative prophage regions in distinct contig sequences (contig accession numbers: BDJA01000001, BDJA01000002, BDJA01000003, BDJA01000004, BDJA01000005, and BDJA01000006) in the genome of strain SCT. The lengths of the putative prophage regions were 11,644 bp (BDJA01000001), 37,073 bp (BDJA01000002), 33,444 bp (BDJA01000003), 5,886 bp (BDJA01000004), 36,693 bp (BDJA01000005), and 18,683 (BDJA01000006). BLASTN best hits in the NCBI database (https://blast.ncbi.nlm.nih.gov/Blast.cgi) using the putative prophage regions as query sequences identified the following taxa: *P. balearica* DSM 6083 (accession: CP007511.1; 75% query coverage and 89% identity) for BDJA01000001, *P. stutzeri* RCH2 (accession: CP003071.1; 4% query coverage and 100% identity) for BDJA01000002, uncultured Caudovirales phage clone 3S_12 (accession: MF417932.1; 70% query coverage and 92% identity) for BDJA01000003, *Pseudomonas aeruginosa* PA7 (accession: CP000744.1; 54% query coverage and 79% identity) for BDJA01000004, *P. stutzeri* DW21 (accession: CP027543.1; 78% query coverage and 90% identity) for BDJA01000005, and *P. stutzeri* 19SMN4 (accession: CP007509.1; 40% query coverage and 94% identity) for BDJA01000006. A total of 184 CDSs were encoded in the six putative prophage regions: 13 CDSs for BDJA01000001 (locus_tag range: PSCT_00201 to PSCT_00214), 54 CDSs for BDJA01000002 (locus_tag range: PSCT_01140 to PSCT_01193), 50 CDSs for BDJA01000003 (locus_tag range: PSCT_01861 to PSCT_01910), 8 CDSs for BDJA01000004 (locus_tag range: PSCT_02604 to PSCT_02611), 35 CDSs for BDJA01000005 (locus_tag range: PSCT_02824 to PSCT_02859), and 24 CDSs for BDJA01000006 (locus_tag range: PSCT_03081 to PSCT_03104). Among the 13 *Pseudomonas* strains analyzed, the largest number of phage-like genes was found in strain SCT with 184, followed by *P. stutzeri* strain 19SMN4 with 109, and then *P. stutzeri* strain CCUG 29243 with 95. These three strains, belonging to the first subgroup of the *P. stutzeri* clade in our phylogeny (Figure 1), were isolated from marine sediments (Table 1). This result is consistent with previous findings reporting the frequent detection and routine isolation of *Pseudomonas* phages in marine environments (Jiang *et al.* 1998; Watkins *et al.* 2018).

Plasmids can confer their hosts resistance to antibiotics and heavy metals (Popowska and Krawczyk-Balska 2013). *P. stutzeri* strains isolated from polluted environments tend to contain plasmids (Ginard *et al.* 1997) and some have been reported to harbor plasmid-encoded silver (Haefeli *et al.* 1984) or mercury resistance (Barbieri *et al.* 1989). *P. stutzeri* strain RCH2 (Chakraborty *et al.* 2017) contained three plasmids pPSEST01, pPSEST02, and pPSEST03 (12,763 bp, 9,865 bp, and 2,804 bp, respectively, in length), and strain 19SMN4 (Rosselló-Mora *et al.* 1994) contained one plasmid, pLIB119 (107,733 bp in length). The four plasmids were not similar to each other based on all-by-all BLASTN searches with a cutoff E value of 1e-5. Given the presence of plasmids in these two strains and their absence in the other *Pseudomonas* strains analyzed, our phylogeny (Figure 1) suggests that these plasmids may have been acquired independently by each lineage, along the branch leading to the ancestor of either strain RCH2 or strain 19SMN4.

### Gene annotations

The genome of *Pseudomonas* sp. strain SCT contained 4,520 CDSs, of which 1,089 are currently annotated as unknown functions (*i.e.*, product names of “hypothetical proteins”), 2,709 are annotated by UniProtKB (Boutet *et al.* 2016), 509 by PFAM (Finn *et al.* 2014), 310 by CDD (Marchler-Bauer *et al.* 2011), and 20 by HAMAP (Lima *et al.* 2009). Of the 4,520 proteins, 4,401 (97.5%) had matches with 4,329 unique records in the UniRef90 database, and 2,543 (56.3%) were assigned to the 1,991 unique KEGG Orthology identifiers. Among the 4,520 CDSs, length in amino acids (Laa) ranged from 30 to 2,842 with a median of 272, and G+C content at the third codon position (GC3) ranged from 33 to 100%, with a median of 79%. G+C content varies more widely at the third codon position than at the first or second positions, which are constrained by protein-coding requirements (Sharp *et al.* 2005). The corresponding data for the SCT genes are shown in Supplementary Table S1.

The following paragraphs detail the functional annotations assigned to SCT genes by Prokka (gene and product names) as well as the UniRef90 and KEGG databases. The SCT genome contains genes encoding cytochrome c and related proteins such as cytochrome c oxidase subunits. A cluster of genes for type II secretion system proteins M, L, K, J, I, H, G, F, and E (locus_tag range: PSCT_03513 to PSCT_03521) was also identified.

Previously, *P. stutzeri* strains were reported as capable of degrading aromatic hydrocarbons, such as phenol, naphthalene, toluene, and xylenes (Rosselló-Mora *et al.* 1994; Nie *et al.* 2016; Brown *et al.* 2017; Singh and Tiwary 2017). Here, the SCT genome appeared to contain genes involved in hydrocarbon degradation, including *benA* and *benB* for large and small subunits of benzoate 1,2-dioxygenase (KEGG: K05549 and K05550); *catA*, which encodes “catechol 1,2-dioxygenase [EC:1.13.11.1]” (KEGG: K03381); and a CDS annotated as “glyoxalase-like domain protein” or “catechol 2,3-dioxygenase” (locus_tag: PSCT_01283). Indeed, some of these genes formed the cluster *benABCDK-catBCA-benE* (contig accession number: BDJA01000005; locus_tag range: PSCT_03059 to PSCT_03067). These results suggest that strain SCT can potentially metabolize aromatic hydrocarbons.

Denitrifying bacteria reduce nitrate (NO_3_^-^) to nitrite (NO_2_^-^), nitric oxide (NO), nitrous oxide (N_2_O), and finally dinitrogen (N_2_) (Carlson and Ingraham 1983). The organization of the denitrification genes has been extensively investigated in *P. stutzeri*, in which *nos* genes (for N_2_O reduction), *nir* genes (for NO_2_^-^ reduction), and *nor* genes (for NO reduction) are adjacent and far from *nar* genes (for NO_3_^-^ reduction) on the chromosome (Baker *et al.* 1998; Arai *et al.* 2003; Yan *et al.* 2005). In the SCT genome, the *nos*, *nir*, and *nor* genes were also adjacent (contig accession number: BDJA01000001; locus_tag range: PSCT_00772 to PSCT_00802) and far from the *nar* genes (contig accession number: BDJA01000010; locus_tag range: PSCT_04176 to PSCT_04184). The gene cluster *narLXK-uspA-narTGHJI-cbf2-moaA-moaB-moeA* (contig accession number: BDJA01000010; locus_tag range: PSCT_04176 to PSCT_04188) is a possible operon consisting of *nar* genes and genes encoding the molybdenum cofactor (Padilla *et al.* 2017). The products of the *moaA*, *moaB*, and *moeA* genes (locus_tag: PSCT_04186, PSCT_04187, and PSCT_04188) are “cyclic pyranopterin monophosphate synthase”, “molybdenum cofactor biosynthesis protein B”, and “molybdopterin molybdenumtransferase”, respectively. The corresponding definitions in the KEGG database (K03639, K03638, and K03750) are “GTP 3’,8-cyclase [EC:4.1.99.22]”, “molybdopterin adenylyltransferase [EC:2.7.7.75]”, and “molybdopterin molybdotransferase [EC:2.10.1.1]”. The genome also contains a cluster of three genes, *moaE-moaD-moaC* (contig accession number: BDJA01000006; locus_tag: PSCT_03192, PSCT_03193, and PSCT_03194), whereby *moaE* encodes “molybdopterin synthase catalytic subunit [EC:2.8.1.12]” (KEGG: K03635), *moaD* encodes “molybdopterin synthase sulfur carrier subunit” annotated also as “molybdopterin converting factor, small subunit” (UniProt: L0GQS7), and *moaC* encodes “cyclic pyranopterin monophosphate synthase [EC:4.6.1.17]” (KEGG: K03637). Molybdopterin is a cofactor essential for nitrate reductase activity (Almeida *et al.* 2017). *Pseudomonas* spp. such as *P. stutzeri*, *P. aeruginosa*, and *P. denitrificans* possess the ability to reduce nitrate, although reduction rates vary among species (Carlson and Ingraham 1983). Strain SCT has been reported to use nitrate or nitrite as an electron acceptor (Amachi *et al.* 2007).

Bacteria, such as *Pseudomonas putida* KT2440 and *P. stutzeri* TS44, possess genes whose products allow resistance to and metabolism of arsenic compounds (Andres and Bertin 2016). We report that the SCT genome contains genes putatively involved in such processes; these include several genes encoding ArsR family transcriptional regulators; *spxA* (locus_tag: PSCT_01329) and *arsC* (locus_tag: PSCT_01343 and PSCT_04450); genes encoding “arsenate reductase [EC:1.20.4.1]” (KEGG: K00537 and K03741), *arsA* (locus_tag: PSCT_02478), which encodes “arsenite/tail-anchored protein-transporting ATPase [EC:3.6.3.16 3.6.3.-]” (KEGG: K01551); and *ubiG* (locus_tag: PSCT_04351), which encodes “arsenite methyltransferase [EC:2.1.1.137]” (KEGG: K07755). There is a cluster of six genes *azr-ywlE-aseR-arsC1-czcO*-CDS (contig accession number: BDJA01000003; locus_tag range: PSCT_01732 to PSCT_01737); of these, *azr* encodes “arsenical resistance protein ArsH” (KEGG: K11811), *ywlE* encodes “arsenate reductase [EC:1.20.4.1]” (KEGG: K03741), *aseR* and *arsC1* encode “ArsR family transcriptional regulator, arsenate/arsenite/antimonite-responsive transcriptional repressor” (KEGG: K03892), *czcO* encodes “putative oxidoreductase CzcO”, and the CDS encodes “arsenite transporter” (KEGG: K03325). These results suggest that strain SCT can potentially resist and metabolize arsenic compounds.

### Gene conservation

Conservation of SCT protein genes in the genome of all *Pseudomonas* strains (Table 1) was determined using the gene screen method with the TBLASTN tool in the LS-BSR pipeline. Of the 4,520 SCT genes, 1,908 (42%) were conserved in all 11 *P. stutzeri* strains, and 1,318 (29%) were conserved in all 13 *Pseudomonas* strains examined. Among *P. stutzeri* strains, more SCT genes were conserved in the three strains belonging to the first subgroup (RCH2, 19SMN4, and CCUG 29243; 3,578 to 3,919 genes) than in the strains belonging to the second subgroup (SLG510A3-8, DSM 4166, CGMCC 1.1803, and A1501; 3,461 to 3,547 genes), or the third subgroup (28a24, 273, and DSM 10701; 2,367 to 2,580 genes). Thus, the conservation of SCT genes in the *Pseudomonas* strains analyzed reflects their phylogenetic relationships (Figure 1).

A total of 451 genes were present in the SCT genome but absent from the other 10 *P. stutzeri* genomes analyzed; they are referred here as the “SCT strain-specific gene set”. They included 254 hypothetical proteins as well as clusters of genes corresponding to putative prophages or possible operons (Supplementary Table S1). The SCT strain-specific genes may have been acquired following separation from common ancestors (gained on the branch leading to the SCT ancestor) and may be associated with SCT strain-specific phenotypic properties (*e.g.*, iodate reduction and living in marine environments).

Sequence statistics (*e.g.*, Laa and GC3) were used to compare SCT strain-specific genes and other genes in the SCT genome. The median Laa value for SCT strain-specific genes (183 aa) was smaller than that for the other genes (280 aa), the difference being highly significant according to a Wilcoxon rank sum test (*p*-value < 2.2e-16). Thus, in general, the SCT strain-specific genes tended to be shorter than the remaining genes in the SCT genome. The median value of GC3 (G+C content at the third codon position) was lower for the SCT strain-specific genes (70%) than for the other genes (79%). Again, the difference was significant according to a Wilcoxon rank sum test (*p*-value < 2.2e-16). Thus, in general, GC3 tended to be lower for the strain-specific genes than for the remaining genes in the SCT genome. For example, GC3 values for a cluster of five genes (contig accession number: BDJA01000002; locus_tag range: PSCT_01674 to PSCT_01678) were lower (ranging from 33 to 41%) than those for the flanking genes (>50%). BLASTP best hits in the UniRef90 database for the flanking genes were identified as belonging to *Pseudomonas* taxa, whereas those for the five genes were unknown (PSCT_01676 and PSCT_01678) or did not belong to the *Pseudomonas* genus; *i.e.*, *Halorhodospira halochloris* (Class: Gammaproteobacteria; Order: Chromatiales) for PSCT_01674, *Inquilinus limosus* (Class: Alphaproteobacteria) for PSCT_01675, and *Alteromonas confluentis* (Class: Gammaproteobacteria; Order: Alteromonadales) for PSCT_01677. Studies have revealed that genes acquired by recent horizontal/lateral transfer often bear unusual nucleotide compositions (Lawrence and Ochman 1997) and are usually rich in A+T (Daubin *et al.* 2003). Thus, nucleotide composition such as total and positional G+C content (GC3) of genes have been used to detect horizontally transferred genes in various complete bacterial genomes (Becq *et al.* 2010).

Some *P. stutzeri* strains are competent for natural genetic transformation (Lorenz and Sikorski 2000), a process whereby DNA is taken up from external environments and is heritably integrated into the genome. This ability enables the bacterium to adapt to various conditions and, not surprisingly, *P. stutzeri* has been found in a wide range of environments (Lalucat *et al.* 2006). Natural transformation of *P. stutzeri* requires a competence phase and the formation of functional type IV pili (Meier *et al.* 2002). Genes for natural transformation (*comA*, *exbB*, and *pil* genes for type IV pili) were found in *P. stutzeri* strains CCUG 29243 (Brunet-Galmés *et al.* 2012) and DSM 10701, a model organism for natural transformation (Chakraborty *et al.* 2017). The SCT genome contains two distinct *pil* gene clusters for type IV pilus assembly protein. One cluster consists of *pilQ-pilP-pilO-pilN-pilM* genes (contig accession number: BDJA01000004; locus_tag range: PSCT_02297 to PSCT_02301; KEGG: K02666, K02665, K02664, K02663, and K02662). Another cluster is SCT strain-specific, and consists of *pilY1-pilX-pilW-pilV-fimT-fimT* genes for “type IV pilus assembly protein” and “type IV fimbrial biogenesis protein FimT” (contig accession number: BDJA01000006; locus_tag range: PSCT_03390 to PSCT_03395; KEGG: K02674, K02673, K02672, K02671, K08084, and K08084). GC3 values for the six SCT strain-specific genes *pilY1-pilX-pilW-pilV-fimT-fimT* were lower (ranging from 53 to 61%) than those for the flanking genes (>73%). Taxa of the BLASTP best hits in the UniRef90 database for the six genes were unknown (PSCT_03390 and PSCT_03392) or did not belong to *P. stutzeri*; *i.e.*, *Microbulbifer agarilyticus* (Class: Gammaproteobacteria; Order: Alteromonadales) for PSCT_03391, *Pseudomonas taeanensis* for PSCT_03393, *Marinimicrobium agarilyticum* (Class: Gammaproteobacteria; Order: Alteromonadales) for PSCT_03394, and *Thiobacillus* (Class: Betaproteobacteria) for PSCT_03395. Evidence suggests that *P. aeruginosa* minor pilins PilV, PilW, and PilX require PilY1 for inclusion in surface pili and vice versa (Nguyen *et al.* 2015). A comprehensive list describing conservation of type IV pili accessory and assembly proteins (FimT, FimU, PilV, PilW, PilX, PilY1, PilY2, and PilE) among *P. aeruginosa* strains was produced by Asikyan *et al.* (2008). Present results suggest that strain SCT may be competent for natural genetic transformation and has been subjected to horizontal gene transfer events via natural transformation.

Extensively studied bacterial resistance systems for mercury are clustered in an operon (*mer* operon), which varies in structure, and consists of genes encoding functional proteins for regulation (*merR*), transport (*merT*, *merP*, and/or *merC*, *merF*), and reduction (*merA*) (Nascimento and Chartone-Souza 2003) of mercury compounds. The marine bacterium *P. stutzeri* 273, which belongs to the third subgroup in our phylogeny (Figure 1), has been shown to be resistant to Hg^2+^ and to be capable of removing it; specifically, genes encoding MerT, MerP, MerA, and MerD appear essential for bacterial mercuric resistance (Zheng *et al.* 2018). SCT strain-specific genes for mercuric resistance/transport/reductase were found in two distinct gene clusters (contig accession numbers: BDJA01000012 and BDJA01000013): *merR-merT-merP-merA-podJ-merR* (contig accession number: BDJA01000012; locus_tag range: PSCT_04381 to PSCT_04386) and *merR-merT-merP-merF-merA-ahpD-comR-dehH2* (contig accession number: BDJA01000013; locus_tag range: PSCT_04402 to PSCT_04409). The first one contains the genes *merR* for “MerR family transcriptional regulator, mercuric resistance operon regulatory protein” (KEGG: K08365), *merT* for “mercuric ion transport protein” (KEGG: K08363), *merP* for “periplasmic mercuric ion binding protein” (KEGG: K08364), *merA* for “mercuric reductase [EC:1.16.1.1]” (KEGG: K00520), *podJ* for “localization factor PodJL”, and *merR* for “mercuric resistance operon regulatory protein”. The second one consists of five *mer* genes (CDS encoding “membrane transport protein MerF” is located between *merP* and *merA*) flanked by *comR*, which encodes “TetR/AcrR family transcriptional regulator, transcriptional repressor for nem operon” (KEGG: K16137) and *dehH2* encoding “2-haloacid dehalogenase [EC:3.8.1.2]” (KEGG: K01560). These results provide genetic evidence of potential mercury resistance and its metabolism by the marine bacterium *Pseudomonas* sp. strain SCT. Moreover, this newly discovered capacity of strain SCT makes the bacterium attractive for the bioremediation of mercury-contaminated environments.

Previous studies have reported the molecular mechanism of 2-haloacid dehalogenase from *Pseudomonas* spp. including *P. putida* (Kawasaki *et al.* 1994; Liu *et al.* 1995; Wang *et al.* 2018) and described known haloacid-dehalogenating bacteria and their dehalogenases (Adamu *et al.* 2016). Of the seven genes annotated as “haloacid dehalogenase” in the SCT genome, three (locus_tag: PSCT_04345, PSCT_04409, and PSCT_04536) were SCT strain-specific. The SCT strain-specific gene (locus_tag: PSCT_04345) encoding “2-haloacid dehalogenase [EC:3.8.1.2]” (KEGG: K01560) is flanked by a CDS (locus_tag: PSCT_04346) encoding “molybdenum cofactor biosynthesis protein A” and *merA* (locus_tag: PSCT_04347), which encodes “mercuric reductase”. Both of these genes are not SCT strain-specific, although the flanking genes *yjlD*-CDS*-oprD-ubiG-pstS1-pstC-pstA-pstB* (locus_tag range: PSCT_04348 to PSCT_04355) are SCT strain-specific.

Bacterial uptake of phosphate occurs via Pi-specific transport (Pst) systems, which are multi-subunit ABC transporters encoded by a four-gene operon, *pstSCAB* (Gebhard *et al.* 2009). A previous study reported the existence of two operons encoding two distinct Pst systems named *pst1* and *pst2* and that *pst2* was present in all *Pseudomonas* spp. including *P. aeruginosa*, *P. fluorescens*, *P. putida*, *P. syringae*, and *P. stutzeri* strains DSM 4166, A1501, and DSM 10701, whereas *pst1* was not (Lidbury *et al.* 2016). The SCT genome contains two *pstSCAB* operons, of which one is SCT strain-specific. The SCT strain-specific *pstSCAB* operon (contig accession number: BDJA01000012; locus_tag: PSCT_04352, PSCT_04353, PSCT_04354, and PSCT_04355) is flanked by *ubiG*, which encodes “ubiquinone biosynthesis O-methyltransferase” also annotated as “arsenite methyltransferase [EC:2.1.1.137]” (KEGG: K07755). Another *pstSCAB* operon (contig accession number: BDJA01000001; locus_tag: PSCT_00287, PSCT_00288, PSCT_00289, and PSCT_00290) is flanked by *phoU* (locus_tag: PSCT_00291), which encodes “phosphate-specific transport system accessory protein PhoU”. Lidbury *et al.* (2016) hypothesized that Pst2 was essential for efficient uptake of Pi in *Pseudomonas* spp. and that Pst1 and Pst2 possessed different kinetic parameters.

Of the 184 proteins encoded on the putative prophage regions in the SCT genome mentioned above, 129 were SCT strain-specific: 3/13 for BDJA01000001, 52/54 for BDJA01000002, 50/50 for BDJA01000003, 8/8 for BDJA01000004, 6/35 for BDJA01000005, and 10/24 for BDJA01000006. The majority (129/184, 70%) of the putative prophage genes are SCT strain-specific. It is likely that the genes in the cluster (*i.e.*, prophage regions) were gained during the same phage transfer event, rather than by several independent events. Our results suggest that phage-mediated gene transfer events have occurred since separation from common ancestors (on the branch leading to the SCT ancestor).

### Conclusion

We report a draft genome assembly for strain SCT, an iodate-reducing bacterium isolated from a marine environment. Phylogenetic analysis indicates that strain SCT belongs to the species *Pseudomonas stutzeri* and is closely related to marine isolates of *P. stutzeri* including strain RCH2. The SCT genome contains genes putatively involved in hydrocarbon degradation, nitrogen metabolism, and arsenic resistance and metabolism. Gene conservation analysis identified a set of genes present in the SCT genome but absent from the other *P. stutzeri* genomes analyzed. This SCT strain-specific gene set included (i) the *pil* gene cluster encoding minor pilins of the type IV pilus system for natural transformation, (ii) *mer* gene clusters encoding resistance systems for mercury, (iii) the *pst* gene cluster encoding Pst systems for uptake of phosphate, and (iv) gene clusters corresponding to putative prophage regions. These results suggest that strain SCT has evolved in marine environments and those polluted by hydrocarbons and heavy metals (*e.g.*, arsenic and mercury) and has been subjected to horizontal gene transfer events via natural transformation and (phage-mediated) transduction. Accordingly, strain SCT has potential in bioremediation of hydrocarbon- and heavy-metal-polluted environments. Finally, bioinformatics analyses of the *Pseudomonas* sp. strain SCT genome sequence have identified a number of new gene targets, whose function will be revealed by future experimental testing.
